# Comparison of breast simultaneous integrated boost (SIB) radiotherapy techniques

**DOI:** 10.1186/s13014-015-0452-2

**Published:** 2015-07-09

**Authors:** Moamen M.O.M. Aly, Gerhard Glatting, Lennart Jahnke, Frederik Wenz, Yasser Abo-Madyan

**Affiliations:** Medical Radiation Physics/Radiation Protection, Universitätsmedizin Mannheim, Medical Faculty Mannheim, Heidelberg University, Mannheim, Germany; Department of Radiotherapy and Nuclear Medicine, South Egypt Cancer Institute, Assiut University, Assiut, Egypt; Department of Radiation Oncology, Universitätsmedizin Mannheim, Medical Faculty Mannheim, Heidelberg University, Mannheim, Germany; Department of Radiation Oncology and Nuclear Medicine (NEMROCK), Faculty of Medicine, Cairo University, Cairo, Egypt

**Keywords:** Breast Cancer, Simultaneous integrated Boost (SIB), Intensity Modulated Radiotherapy (IMRT), Volumetric Modulated Arc Therapy (VMAT)

## Abstract

**Purpose:**

To dosimetrically evaluate different breast SIB techniques with respect to target coverage and organs at risk (OARs) doses.

**Methods:**

Four IMRT techniques were compared in 12 patients. Three techniques employ tangential whole breast irradiation with either two coplanar fields (T-2F), or four non-coplanar fields (T-NC), or one Volumetric Modulated Arc Therapy (T-VMAT) for the boost volume. The fourth technique is a fully-modulated VMAT technique (f-VMAT). Dosimetric parameters were compared for the boost and breast target volumes as well as OARs. Delivery efficiency was analysed based on number of monitor units (MUs) and estimated delivery time.

**Results:**

T-VMAT and f-VMAT ranked highest with respect to integral assessment of boost and breast treatment quality measures. T-VMAT significantly outperformed f-VMAT with respect to ipsi-lateral lung and left-sided patients’ heart volumes ≥ 5 Gy (35 % ± 5 % vs. 52 % ± 6 % and 11 % ± 5 % vs. 22 % ± 6 %, respectively). f-VMAT significantly outperformed T-VMAT with respect to ipsi-lateral lung volume ≥ 20 Gy (13 % ± 2 % vs. 15 % ± 3 %) and heart volume ≥ 30 Gy in left breast cancer (0 % ± 0 % vs. 1 % ± 1 %). T-VMAT and f-VMAT needed 442 ± 58 and 1016 ± 152 MUs, respectively.

**Conclusions:**

The hybrid T-VMAT is considered the technique of choice due to its balance of quality, efficiency and dose to OARs.

## Background

Breast cancer is the most common cancer in women worldwide as it is also the main cause of cancer death among women globally [1]. The use of radiotherapy in the adjuvant setting has shown to improve both local control and overall survival in early stage breast cancer patients [2]. The most common and traditional whole breast radiotherapy technique uses two tangential fields due to its efficiency in terms of sparing nearby organs at risk (OARs) as well as technical simplicity in which wedge filters are used to compensate patient’s surface irregularity and reach a homogenous dose distribution. This technique has evolved over the last decade with the introduction of multi-leaf collimators (MLC) to deliver field-in-field (FIF) three-dimensional conformal (3D-CRT) [3–6] or intensity modulated radiation therapy (IMRT) variants [7–11].

Dose escalation to the tumour-bed by a sequential boost reduces local recurrence [12] but prolongs the treatment duration and significantly increases the risk of moderate to severe breast fibrosis [13]. Alternatively, simultaneously integrated boost (SIB) using a higher dose per fraction to the tumour bed was shown to be dosimetrically advantageous especially regarding dose conformity of the boost volume [14, 15], more convenient due to the shorter treatment time and was recently shown to be very well tolerated on the short and medium terms [16–18].

Different radiotherapy delivery techniques were proposed for SIB, including 3D-CRT with wedges or FIF technique [19, 20], IMRT [21, 22], helical tomotherapy [19], or volumetric modulated arc therapy (VMAT) [23, 24]. A thorough comparison of all these techniques is yet to be performed. In this planning study we compare the dosimetric outcomes of three inversely planned techniques for SIB delivery based on the standard two tangential whole breast fields plus two coplanar boost fields (T-2F), or four non-coplanar boost fields (T-NC), or one boost VMAT arc (T-VMAT) as well as a fully modulated VMAT (f-VMAT) for both the whole breast and integrated boost volumes.

## Materials and methods

### Patient selection and image data

Twelve female breast cancer patients (6 right-sided and 6 left-sided), who were recently treated in the Department of Radiation Oncology, University Medical Centre Mannheim, Heidelberg University, were retrospectively randomly selected. The computed tomography (CT) data-sets were acquired on a CT-simulator (Brilliance CT Big Bore, Philips, Cleveland, OH, USA) according to the institution’s standard protocol in 5 mm slice thickness, in supine position with the use of a wing board for arm positioning above the head.

### Target volumes and organs at risk delineation

Both breast volumes (the affected side, and the contra-lateral breast (CBreast)) were delineated and cropped 5 mm inside the skin contour. Also, the ipsi-lateral lung (ILung), contra-lateral lung (CLung), and heart were delineated. The boost clinical target volume was delineated by an experienced physician according to the scar, pre and post-operative radiological changes within the breast tissue, the surgical report and/or the presence of surgical clips. A setup safety margin of 5 mm was automatically added to this boost volume to create the boost planning target volume (PTV_boost_). This safety margin was constrained to 5 mm behind the skin contour. The whole breast volume subtracting the PTV_boost_ was considered the breast planning target volume (PTV_breast_).

### Beam setup and plan prescription

For each patient, four different IMRT plans were generated using a treatment planning system that employs a Monte Carlo calculation algorithm (Monaco v3.3, Elekta AB, Stockholm, Sweden). A prescribed dose of 64.4 Gy to the PTV_boost_ and 50.4 Gy to the PTV_breast_ in 28 fractions was planned. The plans were created for a 6 MV photon beam Elekta Synergy® linear accelerator with an MLCi2. Except for the VMAT techniques, all other techniques/beams were planned for step and shoot IMRT delivery. Optimization was performed to get the best plan for each technique for each individual patient. The optimization prescription aimed to deliver at least 92 % of the prescribed dose to 95 % of the target volumes and to minimize the volume receiving ≥ 107 % of the boost dose. Having reached these criteria for the targets, additional effort was made to reduce dose to OARs individually for each patient and planning technique starting from the proper choice of gantry angles to the fine-tuning of the prescription cost functions and tightening the constraints to OARs. Our initial planning objectives for the OARs were a mean dose below 5 Gy to the heart for left sided cases, below 3 Gy for contralateral breast and lung, a V_20_ below 22 % for ipsilateral lung. All plans were normalized to deliver a median of 64.4 Gy to the PTV_boost_ volume.

For the first technique, two tangential beams (medial and lateral tangents) were assigned to the PTV_breast_ and another two coplanar oblique beams assigned to the PTV_boost_ with individually selected gantry angles to prevent any unnecessary dose to OARs especially the ipsi-lateral lung. These four fields were optimized together in a single plan (T-2F).

The second technique consisted of the same tangential beams assigned to the PTV_breast_ with four non-coplanar beams assigned to the PTV_boost_ (two gantry angles were chosen for each of two extra couch angles, 45° and 315°) aiming to further reduce OARs exposure, this, as an adaptation from the technique described by Baglan et al. 2003 [25]. These six fields were optimized in a single plan (T-NC).

The third technique was generated by creating a hybrid of tangential IMRT and VMAT deliveries in a single plan by assigning a single VMAT partial arc to the PTV_boost_. The arc typically starts at the same gantry angle assigned for the medial tangential beam and spans (depending on the shape of the thoracic wall, PTV_breast_ and location of the PTV_boost_) to a maximum of 240 °. In this technique, the boost VMAT arc was firstly optimized separately to deliver 14 Gy to the PTV_boost_ and then the resulted plan was used as a biased dose to the tangential plan. The bias-dose option allows loading the dose brought by the VMAT boost arc into the tangential plan to account for it in the optimization of the tangential plan. This strategy leads to the reduction of breast integral dose outside the boost volume. The combined plan was named T-VMAT.

In these three techniques, each beam was assigned to a specific target (i.e. tangential beams to the PTV_breast_ and all other beams or arcs only to the PTV_boost_). Thus, it was possible to prevent the inverse planning system from using the non-tangential beams to target the whole breast which avoids overexposure to the OARs. All these plans were mono-isocentric with the isocentre placed in the structure centre of the PTV_breast_.

The fourth technique was generated using a fully modulated VMAT (f-VMAT) partial double arc, over a maximum span of 240 ° chosen to avoid beam entrance through the contra-lateral organs. In this technique, the isocentre was placed on the centre of the PTV_boost_.

### Plan evaluation

Plan evaluation was based on cumulative dose volume histograms (DVHs). The conformity index (CI) and homogeneity index (HI) for the PTV_boost_ were calculated for each plan using the radiation therapy oncology group (RTOG) definitions [26] according to

1 $$ CI=\frac{V_{\mathtt{64.4}}}{V_{boost}} $$

2 $$ HI=\frac{D_{\mathtt{2}\%}}{\mathtt{64.4}} $$

where *V*_*64.4*_ is the volume of the prescription isodose (64.4 Gy) surface and *V*_*boost*_ is the total boost target volume; *D*_*2%*_ is the dose (Gy) received by 2 % of the boost volume (maximum).

For the PTV_breast_, the quality of coverage (Q) and the heterogeneity index (hI) were calculated using

3 $$ Q=\frac{D_{98\%}}{50.4} $$

4 $$ hI=\frac{D_{2\%}}{D_{98\%}} $$

where *D*_*98%*_ and *D*_*2%*_ are the doses (Gy) received by 98 % (minimum) and 2 % (maximum) of the PTV_breast_, respectively.

Mean dose and volumes above 107 % (V_107_) and below 95 % (V_95_) of the prescribed doses for both target volumes were also compared.

Mean dose and relative volume receiving ≥ 20 Gy (V_20_) of the ILung [27] as well as mean dose and relative volume receiving ≥ 30 Gy (V_30_) of the heart were determined [28]. Furthermore, the mean dose received by the CBreast and CLung were evaluated. To score for prescribed dose and low dose spillage outside the PTV_breast_, two dose spillage indices (DSIs) were used as defined by [23]

5 $$ DS{I}_{50.4}=\frac{V_{50.4}}{V_{\mathrm{PTV}}} $$

6 $$ DS{I}_5=\frac{V_5}{V_{PTV}} $$

where *V*_*50.4*_ and *V*_*5*_ are the volumes of unspecified tissue receiving ≥ 50.4 and ≥ 5 Gy, respectively, and *V*_*PTV*_ is the volume of PTV_breast_.

To account for the low-dose bath and treatment efficiency, OARs relative volumes’ receiving 5 Gy and 10 Gy, the total monitor units (MUs) and the estimated beam-on time (as calculated from the planning system for a maximal dose rate of 600 MU/min) were recorded and analysed for each planning technique.

### Statistical analysis

Descriptive statistics of the data are presented as mean ± standard deviation (SD). The differences of means between the four plans were compared and analysed by a repeated measures one-way ANOVA (with the Greenhouse-Geisser correction and Tukey’s multiple comparisons test) or the Friedman test (with Dunn’s multiple comparisons test) using GraphPad Prism version 6.04 for Windows (GraphPad Software, La Jolla California USA, www.graphpad.com). Statistically significant differences were assumed for a significance level of p < 0.05.

## Results

**Figure 1:**
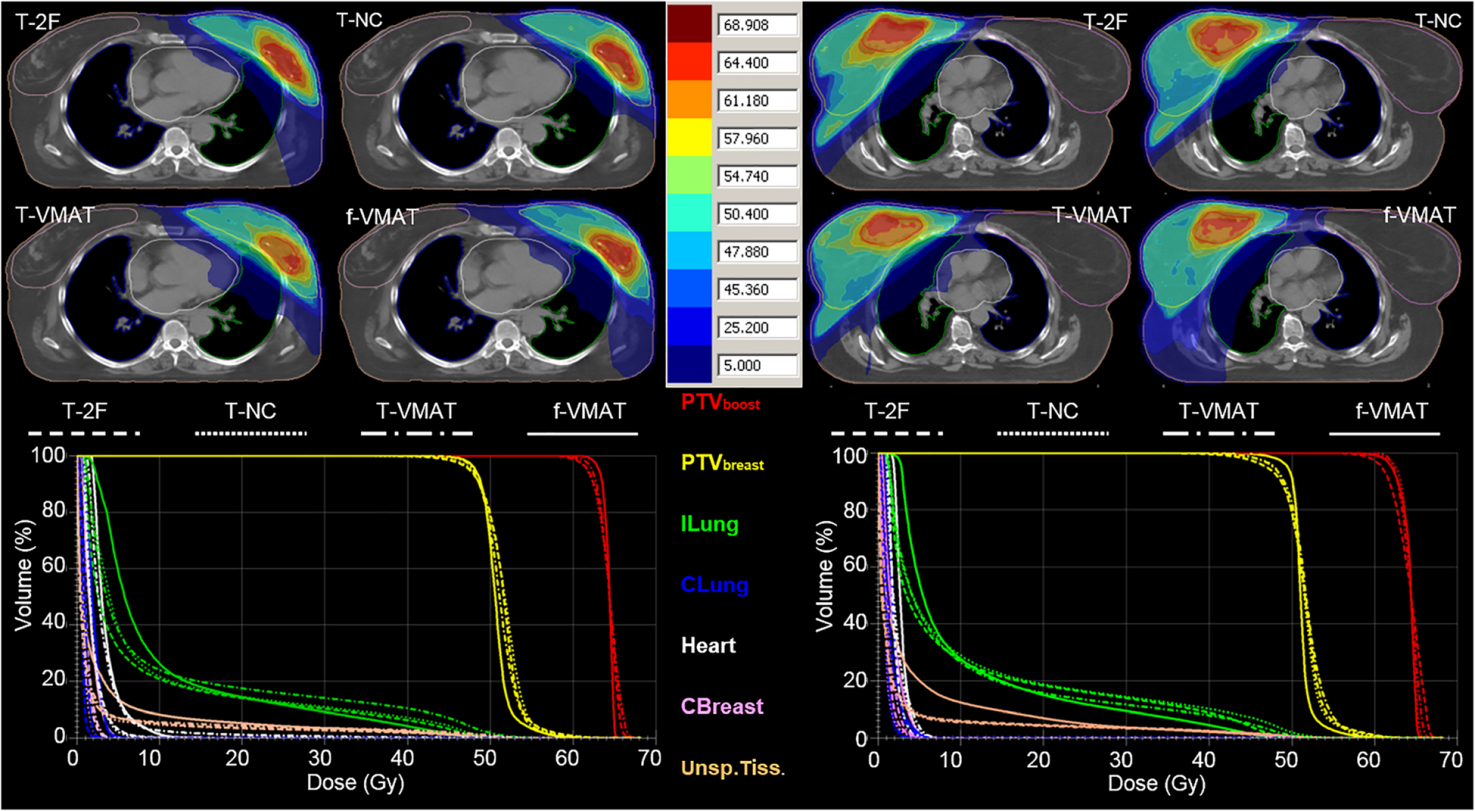
A trans-axial CT slice and the corresponding DVHs of the breast two tangential fields with: two coplanar fields (T-2F), four non-coplanar fields (T-NC), and a VMAT arc (T-VMAT) for the boost volume and a fully modulated VMAT (f-VMAT) techniques for a right-sided (right) and left-sided (left) patient. The DVH line colours correspond to the structure colour

The PTV_boost_ and PTV_breast_ volumes were (46 ± 25) cm^3^ and (1107 ± 401) cm^3^, respectively. Figure 1 shows a trans-axial CT slice with the dose distribution of the four techniques and the corresponding cumulative DVHs for a left-sided and a right-sided breast cancer patient. It demonstrates that the four techniques were able to produce comparable results where the f-VMAT technique achieves the relatively best coverage of the targets.

Table 1 shows the evaluation parameters of the two targets for the studied techniques.V_107_ was equal to zero for PTV_boost_ in all techniques. For PTV_breast_ it ranged from 10.4 % ± 1.3 % in f-VMAT technique to 18.7 % ± 7.1 % in T-NC technique, with the f-VMAT technique being significantly lower than the 3 other techniques. All techniques were able to record a V_95_ ≥ 95 % in PTV_boost_, but the T-2 F recorded the lowest value (95.9 % ± 2.4 %). Both T-2 F and T-NC V_95_ values were significantly lower than that of T-VMAT technique in case of PTV_boost_ and T-2 F V_95_ was also significantly lower than f-VMAT in the two target volumes. The T-VMAT technique was significantly better than other techniques regarding PTV_boost_ mean dose, but not for HI, where the f-VMAT technique was significantly better than other techniques. Regarding the PTV_breast_, the f-VMAT technique was significantly better than the other techniques in mean dose, Q, and hI except for Q in T-VMAT technique where there was no significant difference.

**Table 1:** The targets coverage multi-comparison analysis for all studied techniques (mean ± SD). Mean values with ^ǂ^ symbol demonstrate that the corresponding technique did not pass the normality test. Friedman test of significance (p < 0.05) was used in these cases; otherwise, repeated measures ANOVA significance test was used

N = 12		T-2F	T-NC	T-VMAT	f-VMAT
**PTV**_**boost**_	**V**_**107**_ **(%)**	0^* a^	0^* a^	0^* a^	0^* a^
	**V**_**95**_ **(%)**	95.9 ± 2.4^c^	98.0 ± 0.9^ǂ bc^	99.3 ± 0.6^a^	98.6 ± 1.7^ab^
	**mean (Gy)**	64.1 ± 0.1^b^	64.2 ± 0.0^b^	64.3 ± 0.0^a^	64.1 ± 0.1^b^
	**CI**	0.50 ± 0.00^a^	0.50 ± 0.00^a^	0.50 ± 0.00^a^	0.50 ± 0.00^a^
	**HI**	1.03 ± 0.01^† a^	1.03 ± 0.01^† a^	1.03 ± 0.01^† a^	1.02 ± 0.01^† b^
**PTV**_**breast**_	**V**_**107**_ **(%)**	18.1 ± 3.9^a^	18.7 ± 7.1^ǂ a^	18.7 ± 4.2^a^	10.4 ± 1.3^b^
	**V**_**95**_ **(%)**	94.5 ± 1.8^b^	94.6 ± 1.9^ab^	95.5 ± 2.3^ab^	96.6 ± 1.6^a^
	**mean (Gy)**	52.2 ± 0.3^a^	52.2 ± 0.5^ǂ a^	52.3 ± 0.5^a^	51.5 ± 0.2^b^
	**Q**	0.91 ± 0.02^b^	0.91 ± 0.02^b^	0.92 ± 0.03^ab^	0.94 ± 0.02^a^
	**hI**	1.36 ± 0.03^† a^	1.37 ± 0.04^† a^	1.34 ± 0.05^† a^	1.30 ± 0.03^† b^

^a,b,c^ Values having the same superscript in the same horizontal line are not significantly different.

^*^All the 12 patients have a 0 value.

^†^Note that although these values look similar and have a comparatively small SD (between patients), the differences are significant due to individual patient’s variability (i.e. when looking at the paired data)

V_107_ and V_95_ are volumes receiving 107 % and 95 % of prescribed dose respectively; CI, conformity index as defined by equation (1); HI, homogeneity index as defined by equation (2); Q is the quality of coverage as defined by equation (3); hI, heterogeneity index as defined by equation (4)

Table 2 presents the results of all OARs dosimetric parameters. For ILung mean dose, T-2F was significantly lower than both T-VMAT and f-VMAT, whereas for V_20_ only T-VMAT and f-VMAT showed a significant difference to each other. For left sided cancer patients, the Heart V_30_ was lowest with f-VMAT. Significant differences between the four techniques were found only between T-2F or T-VMAT and f-VMAT. On the other hand, f-VMAT recorded significantly higher mean cardiac dose than all other techniques in right-sided patients and the T-2F and T-NC techniques in left-sided patients. The f-VMAT technique also resulted in higher CLung and CBreast mean doses than the other techniques. The f-VMAT technique revealed the lowest (best) DSI_50.4_ but the highest (worst) DSI_5_ compared to all other techniques where the difference was significant. Differences between the other three techniques were not statistically significant.

**Table 2:** The OARs multi-comparison analysis for all studied techniques (mean ± SD). Mean values with ^ǂ^ symbol demonstrate that the corresponding technique did not pass the normality test. Friedman test of significance (p < 0.05) was used in these cases; otherwise, repeated measures ANOVA significance test was used

N = 12		T-2 F	T-NC	T-VMAT	f-VMAT
**ILung**	**V**_**20**_ **(%)**	14.1 ± 3.5^ab^	13.7 ± 3.5^ab^	14.8 ± 3.4^ǂ a^	13.3 ± 2.4^ǂ b^
	**mean (Gy)**	8.4 ± 1.6^c^	8.8 ± 1.5^abc^	9.1 ± 1.5^ab^	9.5 ± 0.9^ǂ a^
**Heart (Lt.)**	**V**_**30**_ **(%)**	1.3 ± 1.4^a^	1.0 ± 0.9^ab^	1.2 ± 1.4^ǂ a^	0^* ǂ b^
	**mean (Gy)**	3.0 ± 0.9^b^	2.8 ± 0.6^b^	3.5 ± 1.0^ab^	4.2 ± 0.4^a^
**Heart (Rt.)**	**V**_**30**_ **(%)**	0^* a^	0^* a^	0^* a^	0^* a^
	**mean (Gy)**	1.8 ± 0.3^b^	1.6 ± 0.3^ǂ b^	1.9 ± 0.4^b^	2.6 ± 0.2^a^
**CBreast**	**mean (Gy)**	1.1 ± 0.3^c^	0.9 ± 0.2^d^	1.2 ± 0.3^ab^	1.8 ± 0.4^ǂ a^
**CLung**	**mean (Gy)**	0.9 ± 0.2^ǂ bc^	0.7 ± 0.1^c^	1.1 ± 0.2^ab^	1.8 ± 0.3^ǂ a^
**Uns. Tissue**	**DSI**_**50.4**_	0.11 ± 0.05^ǂ a^	0.10 ± 0.05^ǂ a^	0.16 ± 0.07^a^	0.02 ± 0.01^b^
	**DSI**_**5**_	1.42 ± 0.26^b^	1.31 ± 0.19^b^	1.38 ± 0.24^b^	2.65 ± 0.55^a^

^a,b,c,d^ Values having the same superscript in the same horizontal line are not significantly different

^*^All the 12 patients have a 0 value

V_20_ and V_30_ are volumes receiving 20 Gy and 30 Gy respectively; DSI_50.4_ and DSI_5_ are the prescribed (50.4 Gy) and low dose (5 Gy) spillage indexes outside the breast target volume

Concerning all OARs low dose bath, f-VMAT revealed a significantly higher ILung (V_10_ and V_5_) and left-sided patients’ cardiac V_5_ than all other techniques (Table 3).

**Table 3:** The OARs low dose multi-comparison analysis for all studied techniques (mean ± SD). Mean values with ^ǂ^ symbol demonstrate that the corresponding technique did not pass the normality test. Friedman test of significance (p < 0.05) was used in these cases; otherwise, repeated measures ANOVA significance test was used

N = 12		T-2F	T-NC	T-VMAT	f-VMAT
**ILung**	**V**_**5**_ **(%)**	32.0 ± 5.4^c^	37.9 ± 5.2^bc^	35.1 ± 4.6^b^	52.1 ± 6.3^a^
	**V**_**10**_ **(%)**	20.8 ± 4.5^b^	21.0 ± 4.1^b^	21.9 ± 3.9^ǂ b^	25.0 ± 2.7^ǂ a^
**Heart (Lt.)**	**V**_**5**_ **(%)**	6.9 ± 3.4^b^	7.7 ± 3.3^b^	10.9 ± 5.0^b^	22.0 ± 5.6^a^
	**V**_**10**_ **(%)**	3.3 ± 2.6^a^	2.6 ± 1.9^a^	3.6 ± 2.2^ǂ a^	3.3 ± 1.6^a^
**Heart (Rt.)**	**V**_**5**_ **(%)**	0.5 ± 0.3^a^	0.5 ± 0.9^ǂ a^	1.5 ± 1.5^a^	0.7 ± 0.7^a^
	**V**_**10**_ **(%)**	0^* a^	0^* a^	0^* a^	0^* a^
**CBreast**	**V**_**5**_ **(%)**	0.5 ± 0.7^ab^	0.0 ± 0.1^ǂ b^	0.4 ± 0.5^ǂ a^	0.6 ± 0.8 ^a^
	**V**_**10**_ **(%)**	0^* a^	0^* a^	0^* a^	0^* a^
**CLung**	**V**_**5**_ **(%)**	0.2 ± 0.5^ǂ ab^	0.0 ± 0.0^ǂ b^	0.1 ± 0.3^ǂ ab^	0.4 ± 0.5^ǂ a^
	**V**_**10**_ **(%)**	0^* a^	0^* a^	0^* a^	0^* a^

^a,b,c,d^ Values having the same superscript in the same horizontal line are not significantly different

V_5_ and V_10_ are volumes receiving 5 Gy and 10 Gy respectively

Table 4 shows the treatment efficiency parameters for the four techniques. The f-VMAT technique had a significantly higher number of MUs compared to the other techniques. T-NC had a significantly higher estimated beam-on time than f-VMAT.

**Table 4:** Treatment efficiency multi-comparison analysis for all studied techniques (mean ± SD). Mean values with ^ǂ^ symbol demonstrate that the corresponding technique did not pass the normality test. Friedman test of significance (p < 0.05) was used in these cases; otherwise, repeated measures ANOVA significance test was used

N = 12	T-2F	T-NC	T-VMAT	f-VMAT
**MU**	425 ± 72^ǂ b^	411 ± 73^ǂ b^	442 ± 58^b^	1016 ± 152^a^
**Estimated Treatment Time (min)**	3.3 ± 0.7^ab^	3.3 ± 0.6^ǂ b^	2.8 ± 0.5^ǂ ab^	2.9 ± 1.5^ǂ a^

^a,b,c^ Values having the same superscript in the same horizontal line are not significantly different

## Discussion

Exposing the tumour-bed to a higher dose per fraction through SIB in a tumour with a supposedly low α/β ratio [29], has presumably the potential to improve the local control rates while reducing the overall treatment time for patient convenience without increasing side effects in an organ with a low α/β ratio. The possible downside is an increase in breast fibrosis if large volumes are exposed to a high dose per fraction (e.g. when boost volumes are large compared to the breast volume). This puts the focus on finding the optimal SIB technique that adopts a radiobiologically sound dose fractionation schedule through clinical trials [30]. The SIB concept in treating breast cancer was originally presented by Freedman et al. [31] in 2007 showing acceptable cosmetic outcome and quality of life, and was associated with excellent local control rates in 75 treated patients [32]. A more recent early clinical report from a single centre has reported acceptable rates of fibrosis within the boost volume, mostly with very good cosmetic outcomes [16]. Loco-regional control and overall survival rates were also reported to be excellent [18, 20]. Compared to the most widely used sequential electron boost, IMRT-SIB seems to have the advantages of better skin sparing and boost volume conformity especially for deeper boost volumes [14, 33].

Therefore, in search for the optimal breast-SIB radiation technique, we thoroughly examined four modern planning techniques derived from current common practice. In terms of plan quality (homogeneity and conformity), all four techniques fulfilled the basic requirements and are considered applicable and clinically acceptable. However, the assessment of superiority of one technique over the other should be also based on differences in risk of cardiac events, secondary cancers, or fibrosis and treatment delivery efficiency. With the common practice now shifting towards the use of hypo-fractionated schemes with subsequent SIB dose per fraction reaching 3.2 Gy, more focus has to be placed on dosimetric quality of the chosen planning technique to reduce potential side effects [24].

The use of inverse planning with selective targeting of each target volume (selective assigning of tangential beams to the PTV_breast_ and the extra beams to the PTV_boost_) and, when appropriate, the use of biased-dose concept have proven helpful in enhancing the dosimetric qualities of the three tangential based techniques and additionally reduced the differences between them. In comparison to the reported dose values by Scorsetti et al. [24], all our techniques produced lower mean doses to the heart and contra-lateral OARs despite our use of higher total target dose.

The increased risk of cardiac events after cardiac radiation exposure has been the focus of many studies over the past two decades. One of the most current population-based analyses has estimated a linear increase in risk of major coronary events by 7.4 % per 1 Gy increase in the mean radiation dose delivered to the heart [34]. In this context, the f-VMAT technique would be the most unfavourable with the highest mean cardiac dose for right sided breast cancers, although the difference to other techniques was generally not higher than 1 Gy on average. For left sided cancer, only the T-NC was significantly better than f-VMAT (but not compared to the other investigated techniques). Again, the difference in cardiac exposure was not higher than 1.4 Gy on average. Thus, the differences in cardiac risks of all four techniques would be considered minor.

Radiation induced second cancers through whole body exposure to a low-dose-bath (and increased scatter due to higher MUs) with IMRT could also be an issue especially in younger patients. Hall et al. estimated the overall incidence to increase from 1 % after 3D-CRT to 1.75 % with IMRT for patients surviving 10 years [35]. Here, multiple factors come into play: an unconventional increase in the total number of MUs with the subsequent increase in head leakage, higher exposure of many internal organs especially of the contra-lateral breast and lungs, and finally the need for image guided radiotherapy with daily cone-beam-CT (CBCT) for a more accurate patient’s breast set-up. All these factors are directly related to the use of highly modulated planning techniques, especially with intensity-modulated arcs [36, 37]. In our study, we demonstrated the feasibility of keeping the mean dose to the contra-lateral organs below 2 Gy with the largest difference within all four techniques of around 1 Gy on average, which could be considered negligible. Nevertheless, the f-VMAT technique with obviously the highest number of MUs, need for daily CBCT, and significantly higher mean CBreast and CLung doses should preferably be used only in older patients or young patients with challenging anatomy (pectus excavatum, inclusion of parasternal lymph nodes or cardiac contact to the chest wall) where the benefits would outweigh the possible harms [38, 39].

The risk of breast fibrosis is influenced by the use of a boost, of a higher single dose, and the dose per volume [12, 40]. Therefore, special care should be taken to reduce the breast volume (outside the PTV_boost_) that receives doses above the prescription dose. In all four examined techniques, f-VMAT has shown superiority with significantly lower V_107_ and hI.

Regarding treatment efficiency, the estimated beam-on time was around 3 min in all techniques (Table 4). Only T-NC requires practically longer time to apply due to the use of different couch angles.

Another very important aspect is the robustness, i.e. the reproducibility of the prescribed dose distributions. Clearly, the more complex a technique is, the more vulnerable it becomes towards patient, breast setup errors and breathing motions. A tangential IMRT setup was shown to be dosimetrically as robust as conventional wedged fields when a “flash margin” exists. A fully modulated IMRT without a flash margin delivery resulted in under-dosage of the breast surface [41]. Thus, to improve accuracy in the more complex techniques there would be a higher demand to establish sufficient immobilization and setup accuracy through the use of image guidance (e.g. cone-beam CT or surface laser scanners) and/or breath-hold or gating techniques.

The choice of the optimal technique should therefore be performed based on the individual patient’s characteristics; as there is no technique that is best with respect to all criteria. T-VMAT and f-VMAT were in general the best techniques with only a small difference in the mean values. Thus, we recommend the hybrid T-VMAT technique for most of patients. Additionally, being coplanar and tangential based, increases its robustness [42, 43] and reduces low dose spillage outside the target in comparison to f-VMAT with theoretically lower risks of second cancers and cardiac events. T-2F might be the simplest to plan and deliver, but when inversely optimised to avoid dose spikes outside the boost volume (thus being comparable to other multi-beamed techniques) resulted in partial under-dosage within the boost volume. T-NC has the advantage of marginally reducing the low dose spillage thus reducing the mean dose in all OARs. It is however the least practical in terms of delivery and is more demanding in terms of setup accuracy because of the non-coplanar nature of its setup.

## Conclusions

Modern radiotherapy techniques can deliver highly conformal dose distributions and can create different dose levels within the treated volume. The implementation of these advanced modalities needs to be simultaneously optimized with respect to all possibly conflicting treatment goals. Four IMRT breast-SIB techniques with different levels of complexity were explored. While all techniques produced plans of clinically acceptable quality, the VMAT related techniques, f-VMAT and T-VMAT, offered the best target coverage. The T-VMAT would be considered the technique of choice for most patients due to its robustness, practicality and offering a most balanced mix between good target coverage and homogeneity on the one hand versus dose scatter to the OARs on the other hand. For individual cases with left sided cancers and challenging geometry, f-VMAT may be optimal.
